# Associations between Pre-Slaughter and Post-Slaughter Indicators of Animal Welfare in Cull Cows

**DOI:** 10.3390/ani9090642

**Published:** 2019-09-02

**Authors:** Melissa Sánchez-Hidalgo, Carla Rosenfeld, Carmen Gallo

**Affiliations:** 1 Instituto de Ciencia Animal, Facultad de Ciencias Veterinarias, OIE Collaborating Centre for Animal Welfare and Livestock Production Systems—Chile, Universidad Austral de Chile, Valdivia 5090000, Chile; 2 Escuela de Graduados, Facultad de Ciencias Veterinarias, Universidad Austral de Chile, Valdivia 5090000, Chile; 3 Instituto de Medicina Preventiva, Facultad de Ciencias Veterinarias, Universidad Austral de Chile, Valdivia 5090000, Chile

**Keywords:** culled cows, welfare at slaughter, cow health, carcass bruising, carcass condemnations

## Abstract

**Simple Summary:**

Old, sick, and low-production cows are called cull cows, and they are sent from the farms to slaughter to produce meat. Cull cows may be more vulnerable than other categories of cattle regarding alterations of their welfare, but most studies linking animal welfare to meat quality have been carried out in steers and heifers of higher commercial value. In this study, we registered health of cull cows at arrival at a slaughterhouse and handling of the cows during the stunning process and associated those variables with carcass bruising and condemnations; the latter are a reflection of the treatment received by the animals before death and therefore can be used as indicators of animal welfare. We found a high percentage of cull cows arriving with low body condition (skinny), as well as presenting lameness and mastitis. During the stunning process 16% of cows did not fall unconscious after the first shot with the penetrating captive bolt gun. Skinny cows and those with mastitis were more likely to be condemned; skinny cows also presented more severe bruises. We conclude that for cull cows the main animal welfare issue originates at farm level, and cows should be culled sooner.

**Abstract:**

The objective of this study was to evaluate the welfare of cull cows in a slaughtering plant, using indicators of health on arrival and indicators of handling during the stunning process. These pre-slaughter indicators were associated with post-slaughter indicators of the same cows, such as carcass bruising and condemnations. Transport staff surveys showed that all drivers had been trained on animal welfare. All loads of cows came directly from farms and had an average transport duration of 5 h 22 min. Indicators were registered in 237 cows during unloading at the slaughterhouse and in the stunning box. Bruises and condemnations were recorded post-slaughter in the carcasses of the same cows. Results at arrival showed that 48% of the cows had low body condition, 50% had mammary problems, and 24% suffered from lameness. During stunning, 16% of cows needed a second shot, and 54% exceeded the 60 s established as a recommended interval between stunning and bleeding. During the post-slaughter evaluation, 50% of the carcasses had more than two bruises and 70.46% had a bruise severity score different from zero. Low body condition was a risk factor to increase the severity of bruises; low body condition and mammary problems increased carcass condemnations; the stunning process indicators were not statistically associated with the severity of the bruises. For cull cows the main animal welfare issue originates at farm level.

## 1. Introduction

Pre-slaughter handling during the stages of production, transport, marketing, and slaughter can affect animal welfare and also the quality and safety of meat. In cattle, certain characteristics such as the presence of bruises in the carcasses or a high muscular pH, as well as carcass condemnations due to health reasons, can be used as post-mortem indicators of welfare, since they are the result of a lack of welfare in the production system or during the pre-slaughter handling [1,2]. These meat quality problems also cause economic losses to the meat industry [3]. 

Most studies on animal welfare and meat quality in cattle in Latin America, including Chile, have been carried out on steers due to their productive and economic importance [4,5,6,7]. However, there are specific characteristics of certain livestock groups, related to their behavior, age, physical, and physiological condition that make them more vulnerable than others to suffer alterations in their welfare and also cause alterations in the carcasses. One of these groups corresponds to culled cows, which often includes old animals, with low body condition, conformation, or reproductive problems [8]. According to Grandin, cull cows have, in general, low commercial value and do not offer economic incentives to be treated well, hence they are more likely than other cattle categories to suffer from poor welfare [9]. In Chile, cows represent around 20% of all the cattle slaughtered [10].

In Latin America, culled cows are kept longer in lairage giving preference to the slaughter of steers and heifers because they have a higher economic value [11,12]. There is a higher prevalence and also higher severity of bruises in cow carcasses when compared to steer carcasses, particularly if cows are commercialized through livestock auction markets [13,14,15]. Regarding the health of culled cows destined for slaughter, there is scarce information, although it is clear that most condemnations of organs and carcasses occur within this cattle category [16,17]. Moreover, when cows leave the farms, they are often in a poor physical condition and suffering from pain due to illnesses such as lameness and mastitis, which increase the risk of poor welfare during transport and slaughter [18,19,20]. 

The objective of the present study was to carry out an assessment on the welfare state of cull cows in a commercial slaughterhouse using indicators obtained before and after slaughter and to identify possible associations between them.

## 2. Material and Methods

The study was carried out in a meat processing plant in Southern Chile. The methodology included a one-week observation period during commercial slaughter in April 2018. Pre-slaughter indicators of animal welfare were evaluated in cull cows, specifically during the unloading and stunning process, as well as post-slaughter indicators in the carcasses of the same animals, such as bruises or condemnations in order to link them to their health status when they arrived at the slaughterhouse and to the welfare indicators observed during the stunning process.

### 2.1. Animals

Following the methodology of Grandin, who points out that a minimum of 100 animals should be sampled in large slaughter plants [18], a total of 237 cows were observed during the five weekdays of routine slaughter. The criterion to define cull cows was the age, considering all those females with at least four permanent teeth.

### 2.2. Pre-Slaughter Evaluation

#### 2.2.1. Animal Welfare Indicators at Arrival 

The selection criteria for this evaluation was in accordance with the arrival time of the trucks: The animals that were unloaded between 15:00 and 20:00 h were observed every day. During the unloading, a survey was applied to the people in charge of transporting the animals to determine the origin of the cattle (farm or auction market), if the transporter was trained according to the regulations in force, and the travel time. During this stage, the following health indicators were recorded:

Body condition: A value of 0 was assigned to cows with a normal condition, 1 to very skinny, and 2 to very fat, according to the European Welfare Quality protocol [21].

Lameness: Category 0 was assigned to cows without lameness and 1 to cows with lameness; a cow was considered lame if there was an abnormality in its movement, i.e., the animal stands with a flat or arched back, it presents abnormal steps when walking, and the affected limb can be easily identified [21].

Mammary gland problems: Cows presenting an increased volume of one or several mammary gland quarters, a flushed udder suggestive of mastitis, damaged or visibly dry quarters, teats with visible wounds.

Wounds and tegumentary lesions: They were registered as such when the cows presented areas of alopecia in tarsus, hindquarters, carps, neck, shoulders, and back, as well as abrasions, scars, lacerations, and hyperkeratosis, or other type of wounds on their body.

Amputated tail: This was registered when the cows presented visually a shortened tail (less than 50%).

#### 2.2.2. Indicators of Animal Welfare during the Stunning Process

To carry out the evaluation of animal welfare during the stunning process, a follow up was performed in the same cows observed during the unloading process, and the guidelines elaborated by Grandin were used [18,22]. The stunning process was evaluated during the morning hours from 8:00 to 14:00 and the animals observed were those that arrived at the plant the previous day during the afternoon and had been evaluated at unloading; hence, the approximate lairage time was 12 to 18 h, with access to water but no food. The indicators were recorded for each animal entering the stunning box, in the period between the closing of the guillotine door until bleeding. The indicators were recorded as present or absent, and taking as reference the study by Muñoz et al. [23], they were defined as follows:

Hit by the guillotine-type door: When entering the stun box, the cow is hit by the door when this door goes down after activation by an operator.

Vocalization: The cow emits a mooing in the stun box.

Fall: The cow is unbalanced and a part of the body other than the hooves touches the floor of the stun box.

Struggle: While the body, head, and neck of the cow are held by the restraint device inside the stun box, the body of the animal presents sudden movements trying to liberate itself.

Loss of posture at the first shot: The cow falls unconscious after the first shot using the penetrating captive bolt gun.

Time between stunning and bleeding: The period of time between the effective shot and the operator introducing the knife between the first ribs to severe the main blood vessels close to the heart. The Humane Slaughter Association (HAS) [24] (2015) indicates that the interval between firing and bleeding should be kept to a minimum, maximum intervals of 60 s being accepted when using the penetrating captive bolt system. The World Organisation for Animal Health (OIE) [25] (2019) also recommends that in attention to well-being, animals that have been stunned with a reversible method should undergo the bleeding process without delay; when the bovine is stunned with penetrating captive bolt, the maximum period to start bleeding should not be delayed for more than 60 s. Therefore, we defined two classes for the time between stunning and bleeding: ≤60 seg and >60 s.

### 2.3. Post-Slaughter Evaluation

After the death of the animal, two additional aspects were recorded in order to have a complete evaluation of the welfare of the cows in the slaughterhouse:

#### 2.3.1. Condemnations (Condemned Carcasses)

The total or partial condemnation of the carcass of each observed cull cow was recorded if present, as well as the cause of the condemnation, in accordance with the official veterinary inspection carried out by the official veterinarian of the Agriculture and Livestock Service. There were no partial condemnations of the observed carcasses throughout the study, therefore, the condemnation records are understood to be total.

#### 2.3.2. Presence and Characteristics of Bruises in the Carcasses

The observations of the carcasses were made on the slaughter line after the skinning and prior to the separation of the head, an area in which the observation of the entire carcass hanging from the hind limbs is facilitated. The assessment was carried out considering the entire carcass, and the bruises were basically characterized according to the number of them present in each carcass, its extension, and depth as follows:

Number of bruises: The method proposed by European Welfare Quality was used, where the carcass of the cow is valued using three categories: 0 = animal without or with an injury, 1 = animal with a minimum of two lesions and maximum of 10, and 2 = animal with more than 10 lesions [21]. 

Extension of bruises: The evaluation was made by direct observation. We used the Australian Carcass Bruises Scoring System or ACBSS [26] for assessing bruises considering three levels: Extension 1 = from 2 to 8 cm in diameter; extension 2 = from >8 to 16 cm, and extension 3 ≥ 16 cm. Bruises smaller than 2 cm were not considered.

Depth of the bruises: The method described by INE [27] was used. According to the system, the bruises correspond to “grade 1” when the damaged area comprises only the subcutaneous tissue reaching up to the superficial muscular aponeuroses; “grade 2” when the injury affects the subcutaneous and muscular tissues; and “grade 3” involves severe bruises that compromise bone tissue, muscle tissue appears friable with abundant serous exudation, and there is a fresh fracture of the bones in the affected area.

Once the characteristics of the bruises were recorded, a score of severity of bruises was assigned for each observed carcass, adding the points shown in brackets of each characteristic according to Table 1. In this way, the final bruise severity scores ranged between a score of 0, which was awarded to a carcass that did not present any bruise (and hence no extra points were added in terms of extension and depth of bruise), and a score of 8, which was awarded to a carcass with more than 10 bruises (2 points), with an extension of more than 16 cm of diameter (plus 3 points) and a depth of that affected subcutaneous, muscular, and bone tissues (plus 3 points).

### 2.4. Statistical Analysis

The data analysis was performed using the Rstudio statistical program. A descriptive analysis of the indicators evaluated in each of the stages was carried out, and the frequencies (no. and %) of all the indicators were obtained for the total number of cull cows observed. To determine the association between the post-slaughter indicators (condemnations, bruise score) and the pre-slaughter indicators (of both health and welfare during the stunning process), as well as to identify risk factors for condemnations and bruises during the pre-slaughter of cull cows, statistical tests of multinomial logistic regression were carried out.

Prior to data modelling, a univariate analysis was performed in order to select only those variables that were significantly associated with the severity of bruises and carcass condemnations. The most significant variables to be included in the regression model, because they indicated a statistical association between the pre-slaughter and post-slaughter indicators, were the severity of bruises, low body condition, and mammary gland problems. In order to determine the relationship between the bruise severity scores and the preslaughter indicators of health and welfare, a multiple regression analysis was performed, where the dependent variable was the bruise severity score, and the independent variables were the health and welfare indicators. To infer the relationship between carcass condemnations and preslaughter indicators, a generalized lineal model (GLM) was used. The regression analysis performed to study the association between the severity score of the bruises and the variables recorded during the stunning process did not show statistically significant associations, and therefore was excluded from the final model.

## 3. Results

The surveys applied to the transporters showed that the 237 cull cows came directly from the farms and had an average journey length of 5 h 22 min; the shortest journey was 45 min long and the longest one, which consisted of a maritime-terrestrial transport from a farm in the Magallanes Region, lasted 46 h 55 min. During the long journey, the cows received neither water nor food. All the drivers transporting cattle mentioned that they had the training required in Decree 30 of the Chilean Law on the Protection of Animals.

### 3.1. Animal Welfare Assessment According to Pre- and Post-slaughter Indicators

The health indicators of the animals on arrival at the slaughterhouse showed that all the cows with health problems (124) had more than one health problem. Of the 237 evaluated cull cows 52% arrived with a low body condition (CC1), and the predominant health problems were mammary alterations (50%), followed by lameness (24%) (Table 2).

During the stunning process, it was observed that 84% of the cows lost their posture after the first shot, while 16% of the cows needed a second shot since there were signs of sensibility present after the first one. In addition, the interval between stunning and bleeding exceeded 60 s in 54% of the cows. Struggles and vocalizations of the animals were frequently observed in the stun box (Table 2).

In the post-mortem evaluation, it was observed that the condemned carcasses reached up to 7% (16 carcasses), and the reasons for condemnation were massive sarcosporidiosis (9 carcasses), cachexia (5 carcasses), altered organoleptic characteristics (1 carcass), and septic peritonitis (1 carcass).

50% of the evaluated carcasses presented more than 2 bruises, and the extension of the bruises was greater than 2 cm in 54% of the carcasses. The assessment of bruise depth showed that most bruises affected only the subcutaneous tissue (48% of the carcasses), while muscle tissue was affected in 22% of the carcasses and there were no fractures or bruises affecting bone (Table 3).

When calculating the severity score of the bruises as explained in Table 1, it was observed that out of the 237 evaluated carcasses, 70 carcasses (29.53%) obtained a score 0; 13 carcasses (5.49%) had a severity score of 6, and none of the evaluated carcasses reached the highest score (8), which indicates greater severity of bruises in terms of number of lesions present, extension, and depth (Table 4).

### 3.2. Association between Pre- and Post-slaughter Animal Welfare Indicators 

The regression analysis performed to study the association between the severity score of the bruises and the variables recorded during the stunning process did not show statistically significant associations, and therefore were excluded from the final model.

The most significant variables to be included in the regression model, because they indicated a statistical association between the pre-slaughter and post-slaughter indicators (bruises and condemnations), were the severity score of bruises, low body condition, and mammary gland problems.

With a confidence level of 95%, it was established that the risk of having bruises (severity score different from 0) is multiplied by 20 times if the cull cow has a low body condition (1) compared to the optimum of 0 (odds ratio (OR) 20.75; interval of confidence (IC) 2.67–161.42), which means that the cows that arrived at the slaughterhouse with a poor body condition also had more severe bruises in their carcasses (Table 5).

It could be observed that the body condition of the cull cows was a predisposing factor to present more severe bruises in the carcass, therefore, we sought to relate the body condition with other health conditions to find out whether the cows with poor body condition arrived at the plant with other health problems that could lead to a decrease in the level of animal welfare. The results showed that, with 95% confidence, mammary problems (OR 1.77, CI 1.04–3.03) and lameness (OR 1.58, CI 0.84–2.97) were a risk factor to reduce the body condition of the cull cows arriving at the slaughterhouse (Table 6).

A statistically significant association was found between the body condition and the risk of carcass condemnation (*p* < 0.05); in this way, if there was a low body condition (OR 8.61, IC 2.32–55.90) the possibility of carcass condemnation would increase by 8 times. The same happens with mammary problems because if a cow presented mammary problems, the chances of carcass condemnation increased by 4 times (OR 4.29, IC 1.32–19.28) (Table 7).

## 4. Discussion

Previous studies in Chile have shown that carcasses from cows have more bruises than other cattle categories [13,14], which shows poor welfare during the pre-slaughter stages. Moreover, Strappini et al. [28] found that in cows transported for relatively short journeys (<4 h) directly from the farm to the slaughterhouse and similar lairage times (<12 h), most bruises were the result of circumstances at the slaughterhouse. However, this is the first study where the health of cull cows at arrival at the slaughterhouse and the process of stunning were included as pre-slaughter indicators of welfare to be associated with bruising and carcass condemnations as post-slaughter indicators of the cow’s welfare.

### 4.1. Transport

The surveys applied to drivers transporting cattle showed that they all had complied with the training required by the regulations on livestock transport [29]. However, a trip of more than 24 h (46 h) was observed where there was no water or food for the animals, i.e., in breach of said decree. These results agree with a study carried out in Denmark where all the transporters had the certification to carry out this work, however, 41% of them said they did not use the knowledge acquired in the course to correctly transport the animals [30]. Therefore, our results suggest that the training is not being effective with some transporters, and the competent authority should take measures, such as increasing controls to make them comply with the animal welfare regulations.

The proportion of cattle arriving as downers or non-ambulatory can also be used as an indicator of poor welfare. In fact, Grandin and Gallo [31] indicate that some of the most severe problems that occur during cattle transport are with cows for culling. In this sense, it is positive that no non-ambulatory cows were observed at unloading. This indicates that the regulations on transport [29] are being followed, since they declare unfit for transport those animals that cannot stand up on their own, those that have very low body condition or are extremely sick, and females in the last 10% of the pregnancy period.

### 4.2. Pre-Slaughter Health Indicators and Their Association with Bruises and Condemnations

The evaluation of the health indicators in the slaughterhouse showed that the highest percentages of alterations observed in the cull cows corresponded to mammary problems (50%), low body condition (48%), and lameness (24%). Because all culled cows observed had arrived at the slaughterhouse directly from the farms, without passing through auction markets, this finding leads us to conclude that there is a welfare problem at farm level. In agreement with Grandin [18,19], cows are probably pushed beyond their biological limits, and culling is delayed considering only the economically optimal time, which carries a welfare cost. 

The results of the present study are similar to those of a study conducted at a slaughterhouse in Colombia, where 137 cull bovine females presented high percentages of mammary problems, lameness, and skin bruises and carcinoma [17]. In that study, the results were attributed to the lack of good management and timely treatment at farm level and genetic components; all of these health alterations were accompanied by cachexia. These data indicate that the clinical condition of cows at the end of their productive life has not received much attention, although it is highly relevant information to improve the welfare conditions of these animals, as well as fitness for transport and product quality. According to the results of the present study, a faster elimination once the health problem has been detected could improve the welfare of the cull cows in the slaughterhouse and also reduce the condemnations and loss of value of the carcasses, factors which currently do not seem to be considered by the producers.

In our study, as in a similar study conducted in Denmark on cull cows prior to transport [32], we argue that cows are probably not comparable with other younger and healthy cattle, since their fitness for transport is reduced due to age and various clinical findings that may occur. The transport and marketing in a poor state of health and welfare require a greater effort from cull cows than from healthy animals. This is particularly true for lame cows that suffer from pain and are also in low body condition, as they will find it difficult to maintain the balance during walking and especially during transport.

The assessment of body condition is generally used as a measure of nutritional status and, although it does not indicate the current state of hunger, it does provide information on long-term nutritional status [33]. Results similar to those presented in this study were found in cull cows at slaughterhouse level in Colombia, where Romero [17] observed that 98.5% of females that arrived at the establishment had a low body condition. There are similarities with the study conducted in Denmark [32] at the farm level just before the transport to slaughter, where the authors found that 16% of the cows had a poor body condition, concluding that these females should not be considered suitable for transport. Therefore, it is assumed that the low nutritional status of 48% of the cows observed during our investigation was not an acute problem that occurred during transportation, but shows a chronic condition originating at farm level.

The purpose of including the body condition within an evaluation of animal welfare in the slaughterhouse was to identify those cows with some degree of extreme body reserve, since it has been shown that animals with a low or excessive level of body reserve are associated with greater risks of suffering from diseases [34]. In this sense, lameness (OR 1.58, CI 0.84–2.97) and mammary problems (OR 1.77, CI 1.04–3.03) were found to act as risk factors to reduce the body condition of the cows observed. Hence, authors such as Grandin [19] and Losada et al. [35] mention that it is necessary to eliminate the cows before they are in such a poor condition, so that neither their well-being nor their health status are compromised in any extreme way and they don’t become prone to metabolic diseases. In Chile, Decree 30 of the Animal Protection Law states that animals that have a compromised health status in such a way that they cannot stand up by their own means should not be transported [29]. This group often includes cows with poor body condition and severe lameness.

Among the findings related to health, it was observed that 50% of the cull cows had mammary problems, such as inflamed udders, with wounds or lost quarters being the health problem most frequently observed in the slaughterhouse. This result is consistent with a study conducted in Chilean farms, which reports that the incidence rate for mastitis in 2005 was 14.48%, with a slight increase in the winter months [36]. They also agree with Dahl-Pedersen et al. [32], who registered signs of mastitis in one out of five cull cows that were going to be transported from the farm to the slaughterhouse in Denmark. The results of the study also agree with the study carried out in Colombia, where the author reported 20.4% mastitis in cull cows that arrived at the slaughter plant [17]. Dahl-Pedersen et al. [32] point out that mammary problems should not lead to a direct decision of unfit for transport. On the contrary, Grandin mentioned that mastitis is one of the main reasons why cows are not suitable for transport [19]. It has even been shown that relatively mild cases of mastitis are possibly associated with pain [37].

Lameness in cows has been the focus of much scientific attention and is widely recognized as a highly painful condition that leads to the reduction of animal welfare [38]. It is known that lameness in Chile is related to painful diseases such as laminitis, sole ulceration, and digital dermatitis and that it becomes a frequent cause of early culling [39,40]. Therefore, it is reasonable to expect that the arrival of lame cows to the slaughterhouse is a common event and that due to the practices of loading, transport, unloading, and moving they could cause “additional pain”. Thomsen and Sorensen [41] evaluated the pain in cows and reported that transport did not result in changes in the locomotion score of non-lame dairy cows, but this might be different if the cows are lame to some degree before being loaded for transport to markets or the slaughterhouse. To clarify this point, it is necessary to examine the locomotion score in cull cows before and after transport and on arrival at cattle markets.

In a study on the attitudes of farmers and veterinarians towards pain in dairy cows, the participants recognized that mastitis is painful, but no one raised any concerns regarding the suitability for transportation of cows with clinically abnormal udders or mastitis [42]. In Chile, Decree 30 of the Animal Protection Law establishes that very sick animals should not be transported to avoid generating additional pain and suffering [29], however, nothing is mentioned specifically about those cows with mammary or lameness problems. The information on this subject questions whether the current Chilean regulation offers sufficient protection for cows with mastitis, lameness, and other pain related disorders, in addition to suggesting management initiatives to alleviate their condition.

Out of the 237 cull cow carcasses evaluated, 50% had bruises and 7% were totally condemned due to massive sarcosporidiosis, cachexia, septic peritonitis, and altered organoleptic characteristics. The percentage of carcasses with bruises in this study is lower than that found previously in Chile by Strappini et al. [15] and Vargas [43] who used the same protocol and found that 92% of cows observed in a slaughterhouse in Chile, that had been transported directly from the premises, presented bruises. Although there seems to be a reduction in the number of carcasses with bruises over the years, it is necessary to continue working with the staff to achieve improvements in animal welfare and meat quality in cull cows, because the prevalence is still high. The bruises, in addition to being indicative of deficient welfare practices before slaughter, have an economic impact, since the damaged tissue must be cut out of the carcass, generating economic losses. The weight of the extracted tissue decreases the yield of the carcass resulting in reduced financial performance for the producer [44].

The regression analysis performed to determine the association between the severity of the bruises and the pre-slaughter variables showed that the low body condition acted as a risk factor to increase the severity of bruises in the carcass (OR 20.75; IC 2.67–161.42). This agrees with previous work in Chile [14] where it was found that cows with poor body condition have a higher risk of presenting bruises with muscle tissue damage because the presence of fat cover protects the animal from suffering more severe bruises. In Ireland, a study on pigs also found an association between bruises and condemnations with low body condition, bursitis, and tail injuries reflecting a health and welfare problem in the farm [45]. On the other hand, the risk of carcass condemnation in cull cows was also associated with body condition (OR 8.61, IC 2.32–55.90) and mammary problems (OR 4.29, IC 1.32–19.28). This result is consistent with the study carried out in Colombia with culling cows where it was also found that a risk factor for the presentation of bruises and condemnations was body condition (*p* < 0.05) [17]. The results suggest that the poor body condition of the cows upon arrival at the slaughterhouse not only reflects poor animal welfare on the farm (poor nutrition, poor health) but is also a risk factor for condemnation of the carcasses. Moreover, cows in poor body condition need to be handled more carefully because they are at a higher risk of suffering from bruising and causing further detriment in terms of meat quality and quantity.

### 4.3. Indicators of Welfare during the Stunning Process and Their Association with Bruises and Condemnations

During the stunning process, it was found that 16% of the cows did not lose their posture after the first shot, showing a “serious problem” of animal welfare, since this result is far from the 5% accepted by Grandin [18]. This percentage could be improved by providing training to operators, which has been demonstrated in other investigations carried out at slaughter plant level [12,46,47]. The results clearly demonstrate that there is a persistent problem in the stunning process, therefore, it is essential to continue training and regularly monitoring the staff regarding the handling of animals destined for slaughter, as indicated in the regulations [48], in order to improve these indicators and, in turn, the welfare of cull cows.

54% of the cull cows had a period of time between stunning and bleeding longer than 60 s, which is not desirable, since it has been proven that a longer interval (>60 s) favors the recovery of consciousness and the suffering during bleeding, apart from representing a risk for the operator. Furthermore, in terms of meat quality there might be muscle hemorrhages due to the increase in blood pressure [46,49,50]. 

However, the time between stunning and bleeding in this study was shorter than that found in previous research conducted by other authors, such as Gallo et al. [46] in Chile, Romero et al. [51] in a Colombian slaughterhouse, and Sánchez et al. [52] in a slaughterhouse in Ecuador, which indicates that the compulsory training provided to the staff at slaughterhouses in Chile is being partially effective. Sánchez et al. [52] found that 89.60% of the animals presented values higher than one minute between stunning and bleeding, even registering a period of 12 min. On the other hand, in the study conducted in Colombia [51], the average time between stunning and bleeding was 69.53 s. These studies mention the need to train staff to shorten the periods between stunning and bleeding, as it is mainly a problem of lack of knowledge about the importance and consequences of this period on welfare and meat quality.

During the process of stunning, being hit with the guillotine door at the entrance of the stunning box, falls, struggles, and vocalizations, among others are used as indicators within the stunning box that can generate bruises in the carcass and indicate problems of discomfort, stress, and probably pain. Muñoz et al. [23], in a study conducted in Chile, observed that 86.3% of cows were hit with the guillotine door because the stunning operator used this door as a mechanism to accelerate the entry of animals into the box and at the same time to avoid the entry of a second animal. According to the same authors, the animals being hit were mostly dairy cows, due to their large size. This explains why 6.3% of the cows evaluated in this study were hit by the guillotine door. In the study carried out by Muñoz et al. [23], it was found that 4.2% of the cows fell in the stunning box, which was explained in part because the floor was not slip-resistant enough. The analysis of the falls in the present study showed that only 1.2% of cull cows fell within the box, which almost agrees with the maximum 1% accepted by Grandin [18]. The results show that there are some improvements in these indicators over time, although for most indicators there is still a gap to reach the expected standards by Grandin [18].

Vocalizations and struggles in the stunning box are indicators that reflect discomfort or pain in the animal [53]. In this study it was observed that 30% of the cows vocalized when holding the body and the head in the stunning box, and according to the standards to assess animal welfare proposed by Grandin [18], percentages higher than 3% reflect a “serious problem” of animal welfare. In relation to struggle, 32% of the cows presented this indicator during full body restraint inside the stunning box. Our values are higher than those registered by Muñoz and colleagues [23], who recorded that 14.2% of cows vocalized inside the box and 22% of cows struggled, also indicating that these indicators were significantly associated (*p* < 0.001) with a clamping time greater than 5 s. This relationship is explained because the longer the stunning operator delayed stunning after restraining, the more the animal tended to struggle. The high percentage of both indicators in our study probably relate to the fact that in this slaughterhouse a full body plus a head restraint were used and reinforce the need to train the stunning operators and install the possibility of regulating the pressure of the restraint device to help reduce the levels of vocalization, struggle, and discomfort in cows.

The regression analysis performed to determine the association between the severity of the bruises and the variables recorded during the stunning process did not show statistically significant associations. This result differs from those previously reported by Strappini et al. [28] and Muñoz et al. [23], who point out that both the fall of the guillotine door of the stun box on the back of the cattle and the struggles of the animals during the restraint in the box cause bruises. The lack of association is probably due to the lower amount of problems found inside the stun box because the infrastructure currently has non-slip floor, the guillotine doors of the stun box have rubber protections at the base of the doors, and there is also more focus on the good handling of the animals during the training of operators working in this area, which is part of the compulsory training [48].

## 5. Conclusions

There is a welfare problem with cull cows that starts at the farm because the animals arrive at the slaughterhouse presenting a high percentage of health issues, such as lameness, mammary problems, and low body condition, evidencing that their welfare is already compromised before they leave the production site and that there is a lack of a proper culling program at the farm. From our results it can be concluded that training is needed for farmers to ameliorate their awareness about the welfare of cull cows and also the consequences on meat quantity (condemned carcasses) and quality (bruised carcasses).

Low body condition and mammary gland problems act as a risk factor for the severity of bruises and for carcass condemnations, increasing the meat and economic losses in this bovine category.

Animal welfare problems persist during the stunning process, such as the low percentage of cows that fall unresponsive after the first shot and the long period of time between stunning and bleeding as well as several cases of falling and struggling within the stunning box. However, no association was found between these indicators and the animal welfare indicators recorded in the carcasses post-slaughter. Our results clearly suggest that it is essential to regularly monitor these indicators to detect welfare problems in the stunning box and train slaughterhouse staff accordingly in order to improve the welfare of animals for slaughter.

## Figures and Tables

**Table 1:** Variables registered in the post-slaughter evaluation of the cull cow carcasses observed in a meat processing plant in Southern Chile.

Variables	Units (Score)
Number of bruises	Without or with one bruise (0)
	From 2 to 10 bruises (1)
	More than 10 bruises (2)
Extension of bruises	2 to 8 cm (1)
	>8 to 16 cm (2)
	More than 16 cm (3)
Depth of bruises	Subcutaneous tissue (1)
	Subcutaneous and muscular tissue (2)
	Subcutaneous, muscular and bone tissue (3)
Condemnation	Yes (1)
	No (0)

**Table 2:** Descriptive information of ante-mortem animal welfare indicators in 237 cull cows observed in a meat slaughterhouse in Southern Chile.

Variables	Frequencies	
	*N*	%
**Health and welfare indicators**		
Nutritional status		
CC 1	123	52%
CC 0	114	48%
CC 2	0	0%
Amputated tail	33	14%
Wounds and tegumentary lesions	11	4.5%
Lameness	57	24%
Mammary gland problems	119	50.2%
**Indicators during stunning**		
Get hit by guillotine door	15	6.32%
Vocalizations	70	30%
Falls	3	1.27%
Struggles	75	32%
Loss of posture with first shot	199	84%
Time between stunning and bleeding		
≤ 60 s	109	46%
> 60 s	127	54%

**Table 3:** Descriptive information on post-slaughter animal welfare indicators in 237 cull cows observed in a meat-processing plant in Southern Chile.

Variables	Frequencies	
	*n*	%
NUMBER OF BRUISED CARCASSES		
Without or with one bruise	103	43%
From 2 to 10 bruises	119	50%
More than 10 bruises	15	6%
EXTENSION OF BRUISES		
<2 to 8 cm	128	54%
>8 to 16 cm	23	10%
>16 cm	16	7%
DEPTH OF BRUISES		
Subcutaneous tissue	114	48%
Subcutaneous and muscular tissue	52	22%
Subcutaneous, muscular, and bone tissue	0	0%
CONDEMNATIONS		
Total condemnations	16	7%
No condemnations	221	93%

**Table 4:** Descriptive analysis of severity score of bruises observed in 237 cull cow carcasses in a slaughterhouse in Southern Chile.

Bruise Severity Score	Frequency	
	No.	%
0	70	29.53
1	1	0.42
2	24	10.12
3	69	29.11
4	45	18.98
5	15	6.33
6	13	5.49
7	0	0
8	0	0

**Table 5:** Final model of the multinomial logistic regression analysis for the assessment of the severity score of bruises in cull cow carcasses in a slaughterhouse in Southern Chile.

Bruise Severity Score (Dependent Variable)	Independent Variable	OR	IC95%
0	Normal body condition	Ref.	Ref.
1	Low body condition	20.75	2.67–161.42
2		0.57	0.22–1.47
3		0.47	0.24–0.94
4		2.09	0.95–4.59
5		1.42	0.46–4.40
6		0.59	0.18–1.98

OR: Odds ratio; IC: Interval of confidence.

**Table 6:** Final model of logistic regression for the assessment of the association between body condition (dependent variable) and pre-slaughter health indicators (independent variables) of cull cows observed in a meat processing plant in Southern Chile.

Independent variables	Estimate	SD	z Value	*p* Value	OR	95% IC
Lameness	0.45	0.31	1.43	0.15	1.58	0.84–2.97
Mammary gland problem	0.57	0.27	2.12	0.03	1.77	1.04–3.03

SD: Standard deviation; OR: Odds ratio; IC: Confidence interval.

**Table 7:** Final model of logistic regression for the evaluation of the association between condemnations (dependent variable) and pre- and post-slaughter indicators (independent variables) of the cull cow carcasses observed in a slaughterhouse.

Independent Variables	Estimate	SD	z Value	*p* Value	OR	IC
Low body condition score	2.14	0.77	2.79	0.005	8.61	2.32–55.90
Mammary gland problem	1.45	0.66	2.20	0.02	4.29	1.32–19.28
Severity of bruises	0.60	0.45	1.32	0.18	0.54	0.21–1.28

SD: Standard deviation; OR: Odds ratio; IC: Confidence interval.
